# Strong plastid degradation is consistent within section *Chondrophyllae*, the most speciose lineage of *Gentiana*

**DOI:** 10.1002/ece3.9205

**Published:** 2022-08-15

**Authors:** Peng‐Cheng Fu, Shi‐Long Chen, Shan‐Shan Sun, Adrien Favre

**Affiliations:** ^1^ School of Life Science, Luoyang Normal University Luoyang P. R. China; ^2^ Key Laboratory of Adaptation and Evolution of Plateau Biota Northwest Institute of Plateau Biology, Chinese Academy of Sciences Xining P. R. China; ^3^ Senckenberg Research Institute and Natural History Museum Frankfurt am Main Germany; ^4^ Regional Nature Park of the Trient Valley Salvan Switzerland

**Keywords:** *Gentiana*, phylogenomics, plastome, section *Chondrophyllae* s.l., taxonomic treatment

## Abstract

Recovering phylogenetic relationships in lineages experiencing intense diversification has always been a persistent challenge in evolutionary studies, including in *Gentiana* section *Chondrophyllae* sensu lato (s.l.). Indeed, this subcosmopolitan taxon encompasses more than 180 mostly annual species distributed around the world. We sequenced and assembled 22 new plastomes representing 21 species in section *Chondrophyllae* s.l. In addition to previously released plastome data, our study includes all main lineages within the section. We reconstructed their phylogenetic relationships based on protein‐coding genes and recombinant DNA (rDNA) cistron sequences, and then investigated plastome structural evolution as well as divergence time. Despite an admittedly humble species cover overall, we recovered a well‐supported phylogenetic tree based on plastome data, and found significant discordance between phylogenetic relationships and taxonomic treatments. Our results show that *G. capitata* and *G. leucomelaena* diverged early within the section, which is then further divided into two clades. The divergence time estimation showed that section *Chondrophyllae* s.l. evolved in the second half of the Oligocene. We found that section *Chondrophyllae* s.l. had the smallest average plastome size (128 KB) in tribe Gentianeae (Gentianaceae), with frequent gene and sequence losses such as the *ndh* complex and its flanking regions. In addition, we detected both expansion and contraction of the inverted repeat (IR) regions. Our study suggests that plastome degradation parallels the diversification of this group, and illustrates the strong discordance between phylogenetic relationships and taxonomic treatments, which now need to be carefully revised.

## Graphical abstract

*Gentiana* section *Chondrophyllae* sensu lato (s.l.) has undergone evolutionary radiation. Our study shows significant discordance between phylogenetic relationships and taxonomic treatments, and suggests that plastome degradation parallels the diversification of this group.

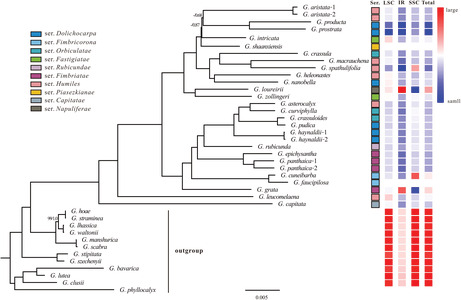

## 1 INTRODUCTION

The increasing availability of plastid genomes represents an excellent opportunity to explore phylogenetic relationships and molecular evolution in plants (Twyford & Ness, 2017). For example, plastid phylogenomics permitted the resolution of some persistent taxonomic uncertainties in challenging plant groups (e.g., Lamiaceae; Zhao et al., 2021), and led to a better understanding of evolutionary patterns both in some selected taxa (e.g., evolutionary radiations in *Saussurea*; Zhang, Landis, et al., 2021) and in major lineages (e.g., Jurassic gap in angiosperms; Li, Ma, et al., 2019; Li, Yi, et al., 2019). Furthermore, comparing plastome structure among related clades and linking the structural changes with diversification can offer clues to the mechanisms driving their evolution (Wicke et al., 2016). In land plants, plastid genomes are generally composed of two inverted repeat (IR) regions that are separated by the large single copy (LSC) region and the small single copy (SSC) region (Jansen & Ruhlman, 2012). Although plastome structure is usually conservative in plants (Mower & Vickrey, 2018), comparative analysis among closely related taxa can provide insights into the evolution of plastid genomes, as for example the expansion/contraction of the IR (Choi et al., 2019; Weng et al., 2017) and gene loss (Lee et al., 2021; Mower et al., 2021; Yao et al., 2019).

Gentians have long attracted the attention of scientists because of their medical, chemical, and horticultural value (Ho & Liu, 2001; Rybczyński et al., 2015). *Gentiana* species are predominantly alpine, and their main center of diversity is located in the Qinghai‐Tibet Plateau (QTP). This area further acted as the primary source for dispersal to many other distant mountainous regions of the world (Favre et al., 2016; Ho & Liu, 2001). Although *Gentiana* is subcosmopolitan, only one species‐rich section (182 species, 51.7% of all *Gentiana*), namely section *Chondrophyllae* Bunge sensu lato (s.l.), is almost globally distributed, whereas another 11 sections of *Gentiana* are endemic to one or two continents (Favre et al., 2016; Ho & Liu, 2001). Section *Chondrophyllae* s.l. is a well‐supported monophyletic group that diverged in the first half of the Miocene (Favre et al., 2016; Fu, Sun, et al., 2021), and includes former sections *Chondrophyllae* Bunge s. str., *Dolichocarpa* T. N. Ho, and *Fimbricorona* T. N. Ho which are intermixed and paraphyletic (Favre et al., 2016, 2020). Often based upon minute morphological traits, section *Chondrophyllae* s. str was divided into 10 series (Ho & Liu, 2001), an example being series *Fimbriatae* Marquand, which is characterized only by filiform calyx lobes and fringed plicae.

Although our understanding of the taxonomy and phylogenetic relationships among Gentianeae genera and within *Gentiana* has greatly improved in the past decades (e.g., Favre et al., 2010, 2020), little is known about the phylogenetic relationship and pattern of molecular evolution in section *Chondrophyllae* s.l. itself, and more specifically among its series. For example, it is unclear whether the intrasectional lineages of section *Chondrophyllae* s.l. are monophyletic. Furthermore, a karyological study revealed varying basic chromosome numbers in the section without any obvious clustering according to series (Küpfer & Yuan, 1996). The phylogenetic relationships within section *Chondrophyllae* s.l. were first studied using internal transcribed spacer (ITS) data, resulting in a poorly supported tree (Yuan & Küpfer, 1997). When more DNA fragments were included, the phylogenetic resolution improved, but the intrasectional relationships were still not resolved (Chen et al., 2021; Favre et al., 2016). Preliminary plastome data showed a great potential to reconstruct a robust phylogeny for section *Chondrophyllae* s.l., although a limited number of species was included (Fu, Sun, et al., 2021). In addition, cytonuclear discordance was observed in section *Chondrophyllae* s.l. (Chen et al., 2021), a possible sign of hybridization, thus showing that maternally inherited DNA might be a promising way to trace the evolutionary history of this group. Furthermore, previous studies have showed that section *Chondrophyllae* s.l. has the most notable plastome size decreases and microstructural changes in the whole subtribe Gentianinae, following gene losses, IR contraction, and SSC reduction (Fu, Sun, et al., 2021). However, these studies did not include all lineages of *Chondrophyllae* s.l. (4 series out of 10). Genome reduction is believed to parallel high evolutionary rate (Wicke et al., 2016) and evolutionary radiations (Kapusta et al., 2017; Moraes et al., 2022). Therefore, more plastomes are needed to verify whether plastome degradation is an ubiquitous trend in section *Chondrophyllae* s.l., and whether it relates to the radiation of this group.

In this study, we newly sequenced plastomes of 21 species belonging to section *Chondrophyllae* s.l., and combined them with existing plastome data in order to reconstruct a robust tree for this group, and assessed whether plastome microstructural changes and current morphology‐based taxonomic treatment are consistent with molecular phylogenetic relationship.

## 2 MATERIALS AND METHODS

### 2.1 Taxon sampling

A total of 21 species (22 individuals) were sampled representing the 10 main series of section *Chondrophyllae* s.l. (Table 1; Table S1). Usually, plants of this section are minute annuals, and thus a whole single plant was collected in the wild for each species, and conserved in silica gel prior to extraction. Species were identified by Dr. Peng‐Cheng Fu and Dr. Adrien Favre, and voucher specimens were deposited either in the herbarium of Luoyang Normal University (no acronym at present), Herbarium Senckenbergianum (FR), or in the Herbarium Universitatis Lipsiensis (LZ). Plant material for two additional species was retrieved from the herbarium of Northwest Institute of Plateau Biology (HNWP) (Table S1).

**TABLE 1:** Plastome structure and sequence information for species of *Gentiana* section *Chondrophyllae* s.l. included in this study.

Species	Taxonomic treatment	GenBank No.	LSC	IR	SSC	Total
*G. haynaldii*	ser. *Dolichocarpa*	MN234137	73,530	22,121	10,117	127,889
*G. haynaldii*	ser. *Dolichocarpa*	ON365620*	73,525	22,130	10,113	127,898
*G. nanobella*	ser. *Dolichocarpa*	ON365616*	74,537	22,892	9754	130,075
*G. producta*	ser. *Dolichocarpa*	MN199163	70,075	19,878	7949	117,780
*G. prostrata*	ser. *Dolichocarpa*	ON365615*	66,620	19,794	8259	114,467
*G. pudica*	ser. *Dolichocarpa*	ON365613*	72,875	22,497	10,328	128,197
*G. cuneibarba*	ser. *Fimbricorona*	MN199137	73,493	22,460	15,164	133,577
*G. faucipilosa*	ser. *Fimbricorona*	ON365602*	‐‐	‐‐	‐‐	100,049
*G. capitata*	ser. *Capitatae*	ON365610*	74,005	22,472	9891	128,840
*G. intricata*	ser. *Fastigiatae*	ON365619*	75,316	23,124	10,177	131,741
*G. zollingeri*	ser. *Fastigiatae*	MZ934753	74,236	22,964	10,598	130,762
*G. epichysantha*	ser. *Fimbriatae*	ON365611*	73,237	21,772	9569	126,350
*G. grata*	ser. *Fimbriatae*	ON365606*	73,965	27,930	5371	135,176
*G. panthaica*	ser. *Fimbriatae*	ON365614*	73,175	21,844	9801	126,664
*G. panthaica*	ser. *Fimbriatae*	ON365605*	73,051	21,680	9744	126,155
*G. aristata*	ser. *Humiles*	MN234139	73,698	22,355	9367	127,775
*G. aristata*	ser. *Humiles*	ON365601*	73,745	22,347	9303	127,742
*G. asterocalyx*	ser. *Humiles*	ON365612*	72,833	22,393	10,238	127,857
*G. heleonastes*	ser. *Humiles*	ON365609*	74,432	23,242	9182	130,098
*G. leucomelaena*	ser. *Humiles*	MT905404	75,476	23,259	9862	131,856
*G. macrauchena*	ser. *Humiles*	ON365604*	73,855	21,718	9327	126,618
*G. spathulifolia*	ser. *Humiles*	ON365607*	71,225	19,423	13,023	123,094
*G. loureiroi*	ser. *Napuliferae*	ON365600*	74,944	29,787	4296	138,814
*G. crassuloides*	ser. *Orbiculatae*	MN199150	73,203	22,370	10,449	128,392
*G. crassula*	ser. *Orbiculatae*	ON365618*	73,526	22,450	9547	127,973
*G. curviphylla*	ser. *Orbiculatae*	ON365608*	72,923	21,941	10,602	127,407
*G. shaanxiensis*	ser. *Piasezkianae*	ON365603*	‐‐	‐‐	‐‐	118,819
*G. rubicunda*	ser. *Rubicundae*	ON365617*	73,045	22,748	10,308	128,849
*G. hoae*	sect. *Cruciata*	MN199141	81,266	25,321	17,084	148,992
*G. straminea*	sect. *Cruciata*	KJ657732	81,240	25,333	17,085	148,991
*G. lhassica*	sect. *Cruciata*	MT982398	80,991	25,304	17,054	148,653
*G. waltonii*	sect. *Cruciata*	MK780032	81,064	25,306	17,029	148,705
*G. manshurica*	sect. *Pneumonanthe*	MT062861	81,347	25,285	17,268	149,185
*G. scabra*	sect. *Pneumonanthe*	MN199131	81,350	25,285	17,269	149,189
*G. stipitata*	sect. *Isomeria*	MG192309	79,712	25,229	16,986	147,156
*G. szechenyii*	sect. *Isomeria*	MN199158	81,581	25,387	16,979	149,334
*G. bavarica*	sect. *Calathianae*	MN199162	80,232	25,468	16,726	147,894
*G. lutea*	sect. *Gentiana*	MN199129	81,815	25,700	17,251	150,466
*G. clusii*	sect. *Ciminalis*	MN199142	80,734	25,566	17,301	149,167

*Note*: Newly sequenced plastomes are indicated with asterisks (*) after the GenBank accession numbers. Columns LSC, IR, and SSC report the length of the large single‐copy, inverted repeat, and small single‐copy regions, respectively, calculated in base pairs.

### 2.2 Sequencing, assembly, and annotation

Total genomic DNA isolation, DNA fragmentation, and sequencing library construction followed the methodology described in Fu et al. (2016). The genomic DNA library of each species was sequenced using the Illumina HiSeq 2500 platform (Novogene), yielding about 2 Gb of 150‐bp paired‐end reads. The plastome was assembled using GetOrganelle v.1.7.1 (Jin et al., 2020) with the default parameters. Each plastid genome was annotated with GeSeq (Tillich et al., 2017) and PGA (plastid genome annotator) (Qu et al., 2019). All plastome sequences were saved as GB2sequin files (Lehwark & Greiner, 2018) and deposited in GenBank (Table 1). In addition to the 22 newly sequenced plastomes, 7 another plastomes in section *Chondrophyllae* s.l. were retrieved from GenBank for downstream analysis (Table 1). Moreover, the entire rDNA cistron was also assembled using GetOrganelle v.1.7.1 (Jin et al., 2020) with the default parameters. The rDNA cistron sequences were deposited in GenBank (ON543454–ON543484) and their details are presented in Table S2.

### 2.3 Phylogenetic analysis

We used the 29 plastomes available in section *Chondrophyllae* s.l. to reconstruct phylogenetic relationships among lineages. Twelve plastomes representing several other sections of *Gentiana* were retrieved from GenBank to server as outgroup (Table 1). Sequences of all protein‐coding genes were extracted in PhyloSuite v.1.2.2 (Zhang, Gao, et al., 2020) and aligned using MAFFT v.7.313 (Katoh et al., 2002). A protein‐coding matrix was constructed where we excluded genes that were absent in some species, or that showed variability that made alignment difficult. We examined the matrix and removed the most rapidly evolving sites using Gblocks v.0.91b (Talavera & Castresana, 2007) using default setting. Phylogenetic analyses were performed with IQ‐TREE v.1.6.8 (Nguyen et al., 2014) implemented in PhyloSuite v.1.2.2 (Zhang, Gao, et al., 2020) using maximum likelihood (ML) and with 1000 rapid bootstrap replicates. The substitution model was chosen using ModelFinder 2 (Kalyaanamoorthy et al., 2017).

Bayesian inference (BI) analysis was run using MrBayes v.3.2.6 (Ronquist et al., 2012). Three runs were started from random trees, with four Monte Carlo Markov Chains (MCMC; one cold and three heated), each for 10 million generations sampling every 1000th. Effective sample sizes (ESS) were well within acceptable values (>200). A majority‐rule consensus tree and posterior probabilities (PP) of bipartitions were computed after 20% of the sampled trees were removed as burn‐in. For rDNA cistron data, ML and BI trees were built following the methodology described above.

### 2.4 Plastome structural changes

Genome comparisons were conducted to identify structural differences using mVISTA (Frazer et al., 2004). The genes on the boundaries of the junction sites of the plastome were visualized in IRscope (Amiryousefi et al., 2018). We tested whether plastome size changes have phylogenetic signal using Pagel's lambda (Pagel, 1997, 1999) in the R package *MOTMOT* (Puttick et al., 2020). *G. faucipilosa* and *G. shaanxiensis* were not included in the phylogenetic signal analysis due to their incomplete plastomes in this study.

### 2.5 Divergence dating

Using the protein‐coding matrix, the divergence times of main lineages were estimated using the Bayesian method implemented in BEAST v.2.4 (Bouckaert et al., 2014; Drummond et al., 2012). We ran the analyses using the Hasegawa–Kishono–Yano (HKY) substitution model, the Yule model, and strict clock model. To improve the accuracy of the molecular dating, we constrained two nodes strictly following the settings in Fu, Sun, et al. (2021). The stem node of *G*. sect. *Cruciata* was constrained with a fossil from the early Miocene (Mai, 2000), using lognormal priors with an offset at 16.0 Ma, a mean of 1.0, and a standard deviation of 1.0. We further constrained the crown age of *Gentiana* using uniform priors with a lower age of 21.25 Ma and an upper age of 38.21 Ma to integrate the entire 95% Highest Posterior Density (HPD) from Janssens et al. (2020). We ran three independent MCMC with 10 million generations, sampling every 1000th generation and discarding the initial 20% as burn‐in. Convergence was judged as suitable by ESS values (>200). Trees were summarized using TreeAnnotator v1.7.5 (Drummond et al., 2012).

## 3 RESULTS

### 3.1 General plastome characteristics

**FIGURE 1:**
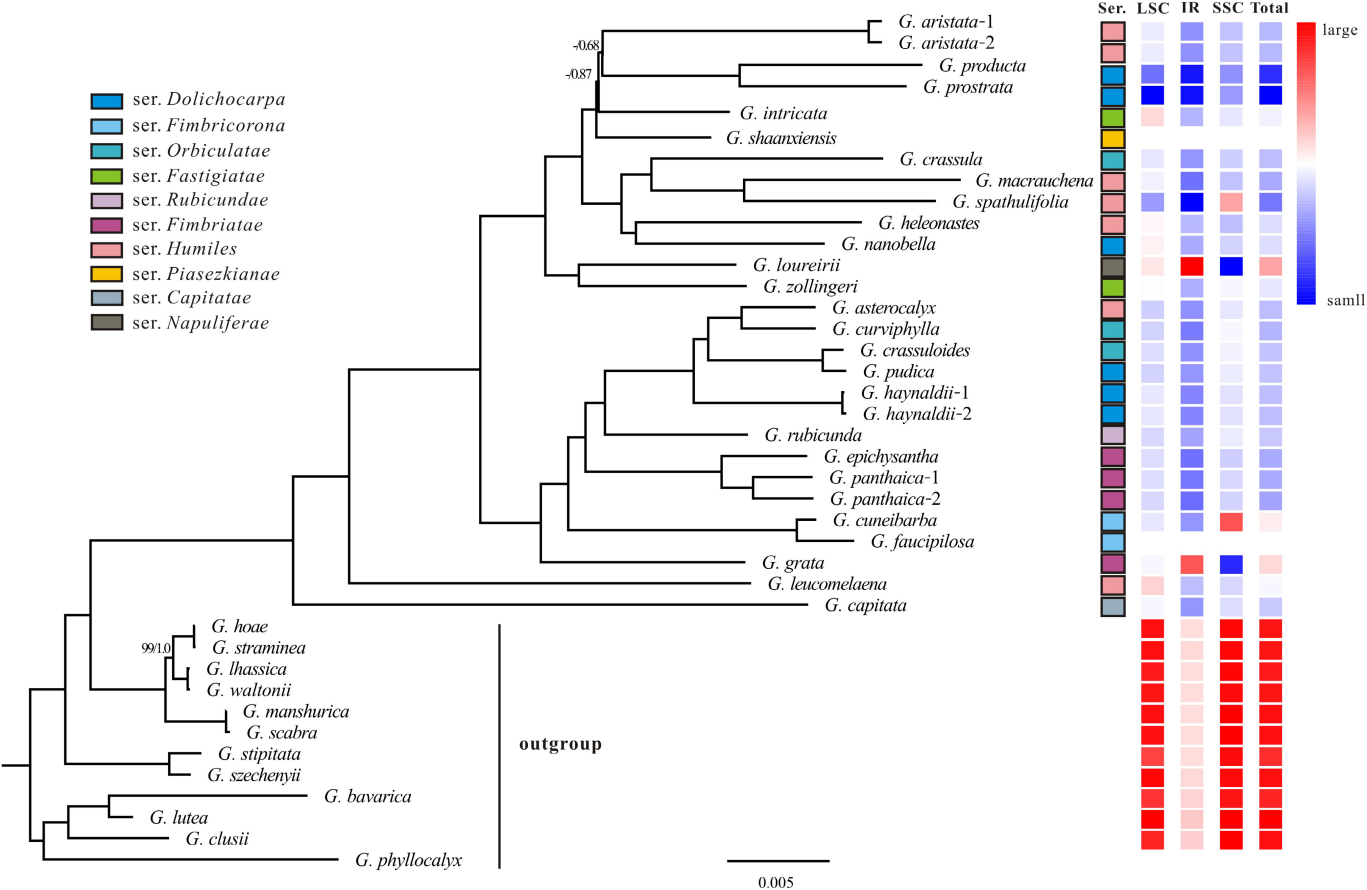
Phylogenetic tree and variation of plastid size in *Gentiana* section *Chondrophyllae* sensu lato. The topology is derived from an analysis of 58 plastid protein‐coding genes. Phylogenetic support values for both maximum likelihood (ML) and Bayesian inference (BI) are shown above branches only when they differ from 100% bootstrap support (BS) and 1.00 posterior probability (PP). Heatmaps illustrate changes in plastid size (LSC, IR, SSC, and total) with relatively reduced sizes in blue and relatively larger sizes in red. The taxonomic attribution of each sample is indicated by colored square with black frame.

In this study, 22 new plastomes representing 21 species of section *Chondrophyllae* s.l. were successfully assembled. Combined with existing plastome sequences, a total of 29 plastomes, representing 26 species which covered the main 10 intrasectional groups of section *Chondrophyllae* s.l. were analyzed in this study. We detected substantial length variation among complete plastomes, with total plastome size varying from 114,467 to 138,814 bp, and with substantial differences in length in the LSC (66,620–75,476 bp), IR (19,423–29,787 bp), and SSC (4296–15,164 bp) (Table 1). The average plastome size of section *Chondrophyllae* s.l. was 128,156 bp, which was much shorter than its closely related sections in *Gentiana* (Figure 1). Similarly, the lengths of LSC, IR, and SSC of section *Chondrophyllae* s.l. were much shorter than those of its closely related sections, except for *G. loureiroi* and *G. grata* which had longer IR. We assembled 7 contigs (from 315 to 75,554 bp) and 14 contigs (from 550 to 25,988 bp) in *G. shaanxiensis* and *G. faucipilosa*, respectively. After mapping to the plastome of *G. haynaldii* (MN234137), we recovered incomplete plastomes of *G. shaanxiensis* and *G. faucipilosa* with their lengths being of 118,819 and 100,049 bp, respectively.

### 3.2 Phylogenetic relationship and divergence time

After filtering, the phylogenetic data matrix included 58 protein‐coding genes shared among all samples. The matrix resulted in a strongly supported topology of section *Chondrophyllae* s.l. (Figure 1). Most nodes, except for two which determined the position of *G. intricata*, were fully supported (bootstrap support value, BS = 100%; posterior probabilities, PP = 1.0) (Figure 1). After *G. intricata* was removed, the support of the uncertain node was improved (BS = 77%, PP = 1.0; Figure S1). We found that *G. capitata* and *G. leucomelaena* were early diverged within section *Chondrophyllae* s.l., which was then further divided into two main clades. The first included six intrasectional groups, namely series *Humiles*, *Fastigiatae*, *Piasezkianae*, *Orbiculatae*, *Napuliferae*, and *Dolichocarpa*. The second clade was a mix of series *Humiles*, *Orbiculatae*, *Rubicundae*, *Fimbriatae*, *Dolichocarpa*, and *Fimbricorona*. Conspecific samples clustered together (three instances), but except for section *Fimbricorona* (*G. cuneibarba* and *G. faucipilosa*), some species belonging to the same intrasectional group did not cluster together (Figure 1). This is, for example, the case for species of series *Humiles*, which are distributed throughout the tree (Figure 1).

**FIGURE 2:**
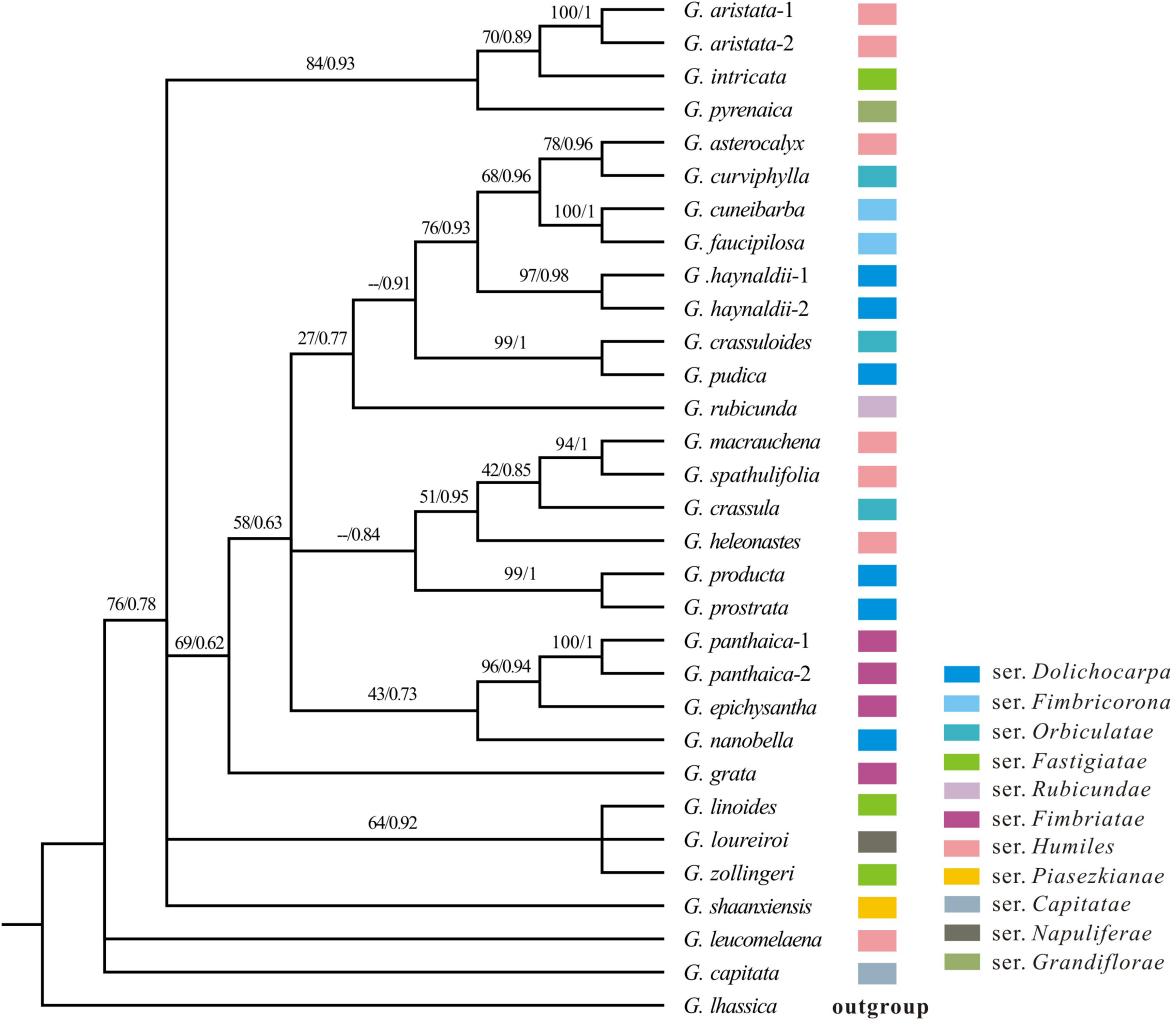
Phylogenetic tree of *Gentiana* section *Chondrophyllae* sensu lato based on recombinant DNA (rDNA) cistron sequences. Numbers on the branches represent bootstrap supports in maximum‐likelihood (ML) analyses and posterior probabilities (PP) in Bayesian inference (BI) analysis. Taxonomic attribution of each sample is indicated by colored square.

A total of 31 rDNA cistron from 28 species (including the outgroup) were assembled in this study (Table S2). Because some rDNA cistrons were not complete, we retained the aligned length of 3545 bp for downstream analyses. The rDNA cistron data resulted in poorly supported ML and BI trees. Although most nodes obtained low support values in both ML and BI trees (Figure 2), the respective backbones of the ML and BI trees were generally consistent with the plastome tree, for example by recovering the early divergence of *G. capitata* and *G. leucomelaena* (Figure S2). Furthermore, as in the plastome tree, rDNA cistron data showed that species from the same series in section *Chondrophyllae* s.l. were not clustered as expected.

**FIGURE 3:**
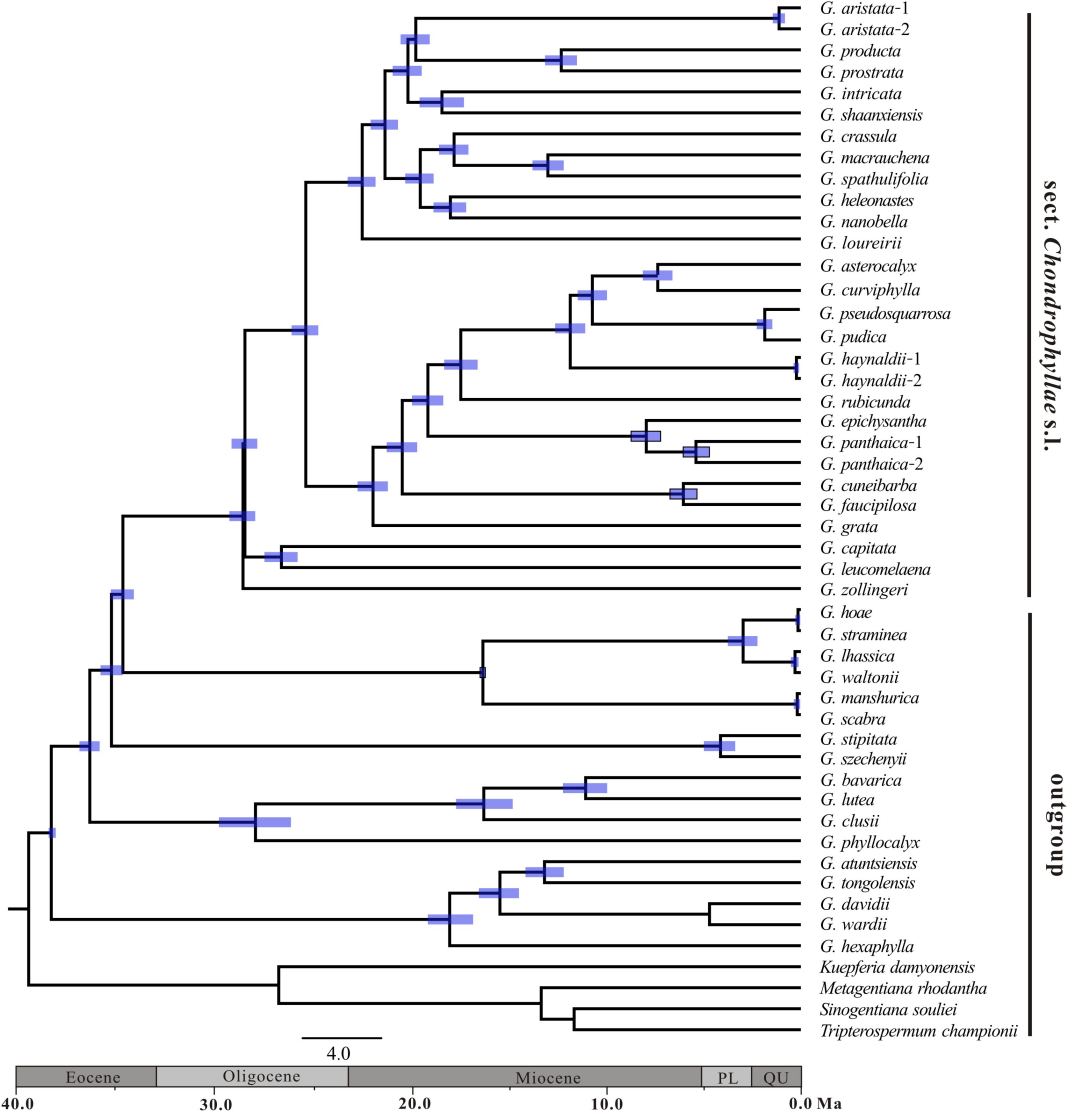
Divergence time estimation in *Gentiana* section *Chondrophyllae* sensu lato. The gray bars show the 95% highest posterior density on the age estimates. The posterior probabilities (PP) of all nodes were 1.0 and are not presented in the figure. Ma, million years ago; PL, pliocene; QU, quaternary.

The divergence time analyses based on plastome data showed that section *Chondrophyllae* s.l. diverged from its sister clade at 34.46 Ma (95% HPD: 33.88–35.05 Ma) (Figure 3). The crown age in *G*. section *Chondrophyllae* s.l. was 28.37 Ma (95% HPD: 27.71–29.03 Ma), corresponding to the second half of the Oligocene. The PP of all nodes were 1.0. The two main lineages in section *Chondrophyllae* s.l. diverged at 25.17 Ma (95% HPD: 24.51–25.87 Ma).

### 3.3 Plastome microstructural changes

**FIGURE 4:**
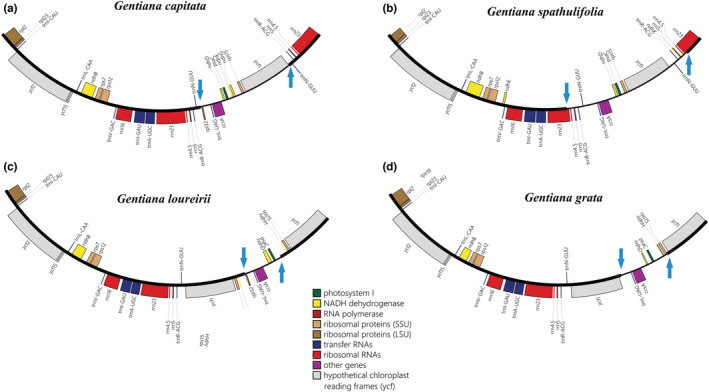
Plastome structural changes in *Gentiana* section *Chondrophyllae* sensu lato (s.l.). (a) *Gentiana capitata* represents a typical IR (inverted repeat)–SSC (small single copy)–IR structure in section *Chondrophyllae* s.l. An IR contraction was detected in *G. spathulifolia* (b). The IR expansion was detected in *G. loureirii* (c) and *G. grata* (d). Genes drawn inside the circle are transcribed clockwise, and those drawn outside are transcribed counterclockwise. Genes belonging to different functional groups are shown in different colors. The blue arrows indicate the boundary of the SSC region.

**FIGURE 5:**
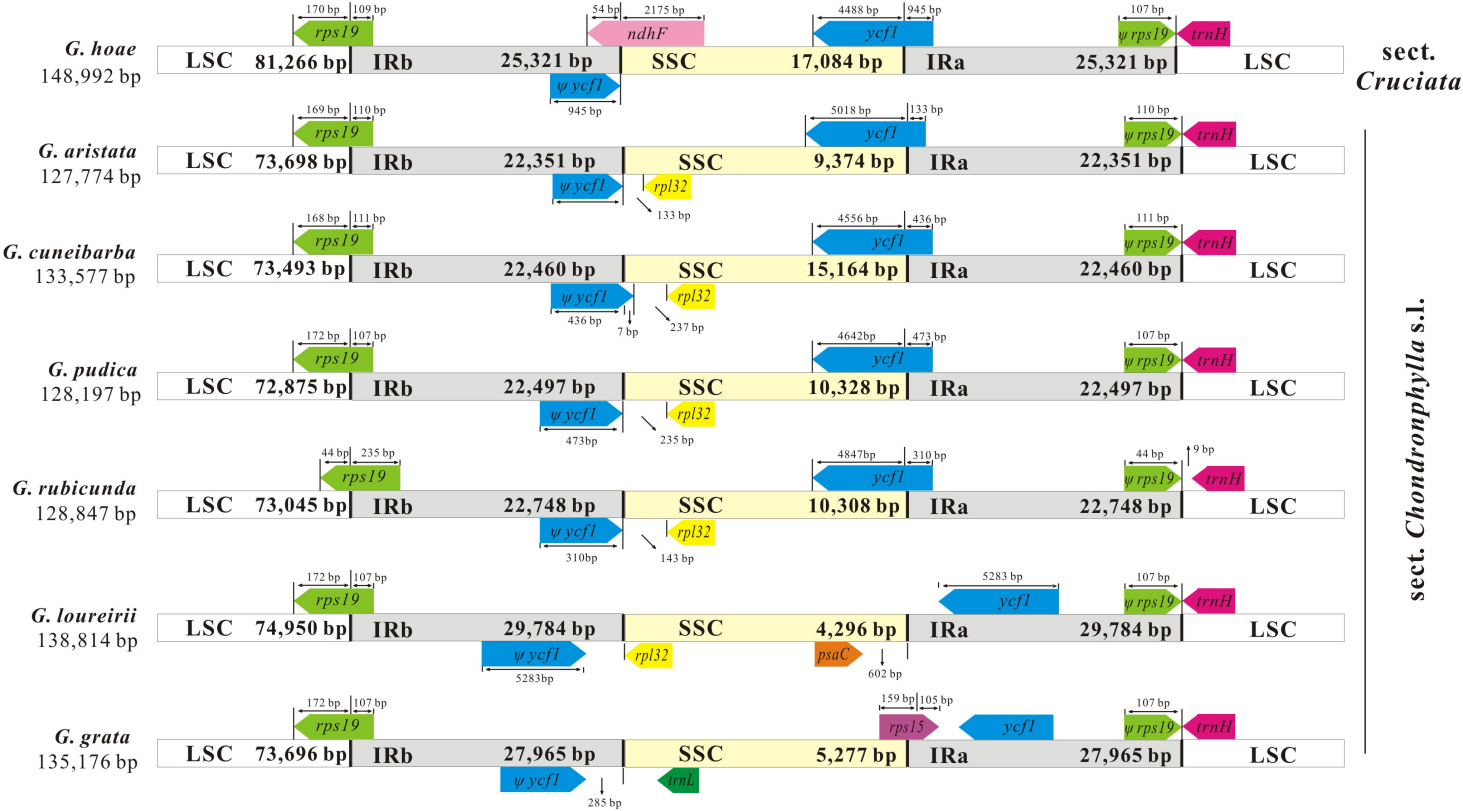
Comparison of large single copy (LSC), inverted repeats (IRs), and small single copy (SSC) junction positions among typical plastomes in *Gentiana* section *Chondrophyllae* sensu lato and closely related sections.

When compared to other closely related sections (e.g., section *Cruciata*), we found that section *Chondrophyllae* s.l. had a similar plastome structure overall. Furthermore, one gene complex (*ndh*) and *rps16*, along with their respective flanking regions, were fully or partly lost in the entire section *Chondrophyllae* s.l., and three introns (*rpoC1* intron, *rpl2* intron, and *clpP* 2nd intron) have been lost in some samples (Figure S3). An expansion of the IR was observed in *G. loureirii* and *G. grata*. In *G. loureirii*, the expansion was caused by the transfer of three plastid genes (*ycf1*, *rps15*, and partial *ndhH*) from the SSC to the IR region (Figure 4). In *G. grata*, the IR expansion was due to the transfer of *ycf1* from the SSC to the IR region. We also observed a contraction of the IR in *G. spathulifolia* due to the transfer of genes (*trnR‐ACG*, *rrn5*, and *rrn4.5*) from the IR to the SSC region (Figure 4). Finally, the contraction of SSC was common in the entire section *Chondrophyllae* s.l., and was due to substantial sequence loss (e.g., *ndh* complex, Figure 4; Figure S3). Various junction site patterns were detected in the plastomes across section *Chondrophyllae* s.l. (Figure 5). The LSC–IRb and LSC–IRa boundaries were relatively stable, while SSC–IRb and SSC–IRa boundaries varied across section *Chondrophyllae* s.l. For example, the SSC–IRa boundary was located within ycf1 across most species, except *G. loureirii* and *G. grata*. The SSC–IRb boundary was not located within *ndhF* as in most other sections in *Gentiana*, but between a pseudogene (*ψycf1*) and *rpl32* or *trnL* in section *Chondrophyllae* s.l. (Figure 5). In addition, tests showed that the ML estimate of Pagel's lambda was equal to 1 for plastome size (LSC, SSC, IR, and total), indicating high phylogenetic signal.

## 4 DISCUSSION

### 4.1 Phylogenetic relationships, taxonomic treatments, and possible reticulate evolution

Recovering the phylogenetic relationships of intensively diversifying taxa has always been a challenging task in evolutionary studies (Olave & Meyer, 2020; Thomas et al., 2021). Using plastome data, we recovered a well‐supported phylogenetic tree and resolved the relationship among the species included in this study with a much improved resolution in comparison to previous molecular studies on section *Chondrophyllae* (Chen et al., 2021; Favre et al., 2016; Yuan & Küpfer, 1997). The phylogenetic power of our study, harnessed from genomic data, thus echoes that reported in an increasing number of similar investigations on the evolutionary history of radiating alpine taxa, such as *Rhodiola* (Zhao et al., 2020) and *Saussurea* (Zhang, Landis, et al., 2021; Zhang, Yu, et al., 2021).

Furthermore, we found that the currently recognized taxonomic treatment within section *Chondrophyllae* s.l. (e.g., Ho & Liu, 1990) is relatively inconsistent with phylogenetic relationship we recovered in the trees based on plastome and rDNA cistron sequences (Figures 1 and 2). For example, the two better‐sampled groups in our study, namely series *Dolichocarpa* and *Humiles*, were not monophyletic (Figure 1). Although the number of *Chondrophyllae* s.l. species included in this study is limited (26 out of ca. 180), we believe increasing the number of samples would not recover monophyletic clades for series *Dolichocarpa* and *Humiles*, given that all other known main lineages within the section were included. Also, the results of other studies showed the same pattern (Chen et al., 2021), including with Sanger sequencing with a much higher proportion of species (Favre et al., 2016). Reticulate evolution is likely to be a major contributor to this inconsistent pattern, as well as to an accelerated diversification. Reticulate evolution was also suggested in *Swertia*, another species‐rich genus of Gentianeae in the Tibeto‐Himalayan region (Chassot et al., 2001), as well as in other taxa such as woody bamboo (Guo et al., 2021), *Lachemilla* (Morales‐Briones et al., 2018), and even lizards (Esquerré et al., 2022). Hybridization is at the source of reticulate evolution, and in *Gentiana*, interspecific crosses were detected in several sections in both the region of the Qinghai‐Tibet Plateau (QTP) and Europe (Favre et al., 2021; Fu, Twyford, et al., 2021; Hu et al., 2016). However, no direct evidence of hybridization was ever reported in section *Chondrophyllae* s.l., although cytonuclear discordances in the phylogeny produced in this study, as well as other evidence based upon transcriptome data (Chen et al., 2021) suggest that hybridization could be common also in this group. In fact, current and past hybridization events are only poorly investigated as potential contributor to diversification in the alpine biome of the region of the QTP. This shortcoming is for example most visible in *Saxifraga*, for which it was reported that hybridization was intense in Europe and almost absent in the region of the QTP (Ebersbach et al., 2020). In summary, evidence suggests that the current taxonomic treatment within section *Chondrophyllae* s.l. needs to be revised with the help of advanced molecular data and an increased species cover, and that the extent of past and present events of hybridization should be evaluated in this section.

### 4.2 Is plastome degradation related to radiation?

Plastome degradation is visible as the loss of genes and sequences, and was observed in a wide range of vascular plant lineages (Lehtonen & Cárdenas, 2019; Mohanta et al., 2020; Yao et al., 2019). Having sampled all main morphological lineages of section *Chondrophyllae* s.l., our study identified a strong and consistent plastome degradation in this group. Indeed, section *Chondrophyllae* s.l. displays the shortest average plastome sizes (128 Kb) in Gentianeae, as sister subtribes Gentianinae and Swertiinae were found to have plastome sizes ranging from 135 to 151 Kb (Fu, Sun, et al., 2021) and from 149 to 153 Kb (Zhang, Sun, et al., 2020; Zhang, Yu, et al., 2021), respectively. Shorter plastomes in this case are due to structural changes such as SSC contraction and frequent gene losses, but did section *Chondrophyllae* s.l. experience rapid diversification or even explosive radiation?

First, we need to keep in mind that species of section *Chondrophyllae* s.l. are usually characterized by long branches in phylogenetic trees (Figure 1; e.g., Fu, Sun, et al., 2021). Hence, this clade has accumulated many more genetic modifications than closely related lineages in the same lapse of time, suggesting a higher molecular evolution than other sections in *Gentiana*. This is indirectly supported by the sheer number of *Chondrophyllae* species (representing 51.7% of all species in the genus, i.e., 182 species; Ho & Liu, 2001; Favre et al., 2020), and by a reported accelerated substitution rate, admittedly using a limited sampling (Fu, Sun, et al., 2021). Second, it was reported that accelerated substitution rates may be associated with plastome size (Schwarz et al., 2017) and life history (e.g., annual vs. perennial; Gaut et al., 2011), and in fact, most species of section *Chondrophyllae* s.l. are annual, with the exception of series *Napuliferae*. This series is one of the two species‐poor series in the section, containing only three species (Ho & Liu, 2001), and interestingly, series *Napuliferae* has also the longest plastome (*G. loureirii*, 138 Kb) in section *Chondrophyllae* s.l. (based upon currently available data). Thus, it seems that plastome degradation may be correlated with the life cycle and diversification rates in section *Chondrophyllae* s.l., as suggested by (Fu, Sun, et al., 2021) and observed in other taxa such as Orchidaceae (Li, Ma, et al., 2019; Li, Yi, et al., 2019; Tang et al., 2021). Nevertheless, because some plastome degradation was also observed in a few perennial lineages of Gentianinae (Fu, Sun, et al., 2021; Sun et al., 2018) and other perennial plant lineages (e.g., Tang et al., 2021; Zhou et al., 2022), more species of section *Chondrophyllae* s.l. need to be investigated to understand fully whether this lineage has undergone explosive radiation. In any case, the diversification of section *Chondrophyllae* s.l. may have been fostered by the climatically and geologically dynamic context of the region of the QTP. As stated by the “*Mountain‐Geobiodiversity Hypothesis*” (Mosbrugger et al., 2018), a species‐pump effect is likely to have been a powerful driver of diversification in this region. Indeed, it would be expected that such climate‐driven cycles of range expansions and contractions, alternatively forcing allopatry and secondary contacts among closely related (and possibly interfertile) taxa, may have disproportionately affected the diversification of annuals in comparison to perennials. This, however, remains yet to be tested in section *Chondrophyllae* s.l. and across multiple taxa.

### 4.3 Conclusion

By sampling the main evolutionary lineages in *Gentiana* section *Chondrophyllae* s.l., we have discovered a consistent plastid degradation in the entire clade, including the loss of functional genes and sometimes short single‐copy regions. Whether or not section *Chondrophyllae* s.l. experienced explosive radiation is still partially up for debate, although several lines of evidence (including short plastomes) indicate that it might be the case. A taxonomic revision will be necessary to further understand the mechanisms involved in the evolutionary history of section *Chondrophyllae* s.l., including hybridization within a context of rapidly changing geological and climatic settings during the last few million years.

## AUTHOR CONTRIBUTIONS

**Peng‐Cheng Fu:** Data curation (equal); methodology (equal); resources (equal); software (equal); writing – original draft (equal). **Shilong Chen:** Data curation (equal); investigation (equal). **Shan‐Shan Sun:** Funding acquisition (equal); project administration (equal); visualization (equal); writing – review and editing (equal). **Adrien Favre:** Funding acquisition (equal); resources (equal); writing – review and editing (equal).

## CONFLICT OF INTEREST

None declared.

## Supporting information


Table S1

Table S2

Figure S1

Figure S2

Figure S3

## Data Availability

All data are provided within the text, tables, figures and supplements.
