# Supplementary material for: Strong plastid degradation is consistent within section Chondrophyllae, the most speciose lineage of Gentiana
Source: Ecol Evol. 2022 Aug 15;12(8):e9205. doi: 10.1002/ece3.9205 (PMC9379351; doi:10.1002/ece3.9205)
Supplement: Supplementary file 1 — Table S1 [file ECE3-12-e9205-s005.docx]

**APPENDIX A**

Table A1 Information of samples sequenced in this study.

| Species | Author | Collection No. | Location | Country | Deposition location |
| --- | --- | --- | --- | --- | --- |
| *G. aristata* | Maximowicz | AFCN_18008 | South of Xining, Qinghai | China | FR |
| *G. asterocalyx* | Diels | AFCN_11106 | Lijiang, Yunnan | China | LZ |
| *G. capitata* | Buchanan-Hamilton ex D. Don | HNWP-81750 | Jilong, Tibet | China | HNWP |
| *G. crassula* | Harry Smith | AFCN_11134 | Baishuitai, Yunnan | China | LZ |
| *G. curviphylla* | T. N. Ho | AFCN_11220 | Litang, Sichuan | China | LZ |
| *G. epichysantha* | Handel-Mazzetti | AFCN_11131 | Bashuitai, Yunnan | China | LZ |
| *G. faucipilosa* | Harry Smith | Fu2018068-7 | Gongshan, Yunnan | China | LY |
| *G. grata* | Harry Smith | AFCN_11032 | Bingzhongluo, Yunnan | China | LZ |
| *G. haynaldii* | Kanitz | AFCN_11194 | Daocheng, Sichuan | China | LZ |
| *G. heleonastes* | Harry Smith | AFCN_11206 | Yading, Sichuan | China | LZ |
| *G. intricata* | C. Marquand | Fu2020081-1 | Lijiang, Yunnan | China | LY |
| *G. loureiroi* | (G. Don) Grisebach | Fu2016220-10 | Guidlong, Hunan | China | LY |
| *G. macrauchena* | C. Marquand | Zhengb096 | Shennongjia, Hubei | China | LY |
| *G. nanobella* | C. Marquand | AFCN_11179 | Baimaxuashan, Yunnan | China | LZ |
| *G. panthaica* | Prain & Burkill | LP174134 | Shennongjia, Hubei | China | LY |
| *G. panthaica* | Prain & Burkill | AFCN_11061 | Dali, Yunnan | China | LZ |
| *G. prostrata* | Haenke | Mosquin PM 26 | unknown | unknown | LZ |
| *G. pudica* | Maximowicz | AFCN_11176 | Baimaxueshan, Yunnan | China | LZ |
| *G. rubicunda* | Franchet | Zhengb098 | Shennongjia, Hubei | China | LY |
| *G. shaanxiensis* | T. N. Ho | HNWP-71659 | Ningqiang, Shaanxi | China | HNWP |
| *G. spathulifolia* | Maximowicz ex Kusnezow | AFCN_11304 | Songpan, Sichuan | China | LZ |
| *G. zollingeri* | Fawcett | LP161491 | Kuangcheng, Hebei | China | LY |
