# Supplementary material for: Strong plastid degradation is consistent within section Chondrophyllae, the most speciose lineage of Gentiana
Source: Ecol Evol. 2022 Aug 15;12(8):e9205. doi: 10.1002/ece3.9205 (PMC9379351; doi:10.1002/ece3.9205)
Supplement: Supplementary file 2 — Table S2 [file ECE3-12-e9205-s001.docx]

Table A2 Information of rDNA cistron sequences assembled in this study.

| Organism | Specimen_Voucher | GenBank no. | Raw data inference |
| --- | --- | --- | --- |
| *G. aristata* | AFCN_18008 | ON543454 | this study |
| *G. aristata* | Fu2017031-3 | ON543455 | Fu et al. 2021a |
| *G. asterocalyx* | AFCN_11106 | ON543456 | this study |
| *G. capitata* | HNWP-81750 | ON543457 | this study |
| *G. crassula* | AFCN_11134 | ON543458 | this study |
| *G. crassuloides* | Fu2016031-10 | ON543459 | Fu et al. 2021a |
| *G. cuneibarba* | Fu2018089-1 | ON543460 | Fu et al. 2021a |
| *G. curviphylla* | AFCN_11220 | ON543461 | this study |
| *G. epichysantha* | AFCN_11131 | ON543462 | this study |
| *G. faucipilosa* | Fu2018068-7 | ON543463 | this study |
| *G. grata* | AFCN_11032 | ON543464 | this study |
| *G. haynaldii* | AFCN_11194 | ON543465 | this study |
| *G. haynaldii* | Fu2018166-1 | ON543466 | Fu et al. 2021a |
| *G. heleonastes* | AFCN_11206 | ON543467 | this study |
| *G. intricata* | Fu2020081-1 | ON543468 | this study |
| *G. leucomelaena* | Miao1902 | ON543469 | Ya et al. 2020 |
| *G. linoides* | AFCN_18161 | ON543470 | Favre et al. 2020 |
| *G. loureiroi* | Fu2016220-10 | ON543471 | this study |
| *G. macrauchena* | Zhengb096 | ON543472 | this study |
| *G. nanobella* | AFCN_11179 | ON543473 | this study |
| *G. panthaica* | LP174134 | ON543475 | this study |
| *G. panthaica* | AFCN_11061 | ON543474 | this study |
| *G. producta* | Fu2017242-2 | ON543476 | Fu et al. 2021a |
| *G. prostrata* | Mosquin PM 26 | ON543477 | this study |
| *G. pudica* | AFCN_11176 | ON543478 | this study |
| *G. pyrenaica* | Kozuharova_Rila_Mts | ON543479 | Favre et al. 2020 |
| *G. rubicunda* | Zhengb098 | ON543480 | this study |
| *G. shaanxiensis* | HNWP-71659 | ON543481 | this study |
| *G. spathulifolia* | AFCN_11304 | ON543482 | this study |
| *G. zollingeri* | LP161491 | ON543483 | unpublished |
| *G. lhassica* | Fu2016204 | ON543484 | Fu et al. 2021b |
