# Supplementary figures and images for: Strong plastid degradation is consistent within section Chondrophyllae, the most speciose lineage of Gentiana
Source: Ecol Evol. 2022 Aug 15;12(8):e9205. doi: 10.1002/ece3.9205 (PMC9379351; doi:10.1002/ece3.9205)

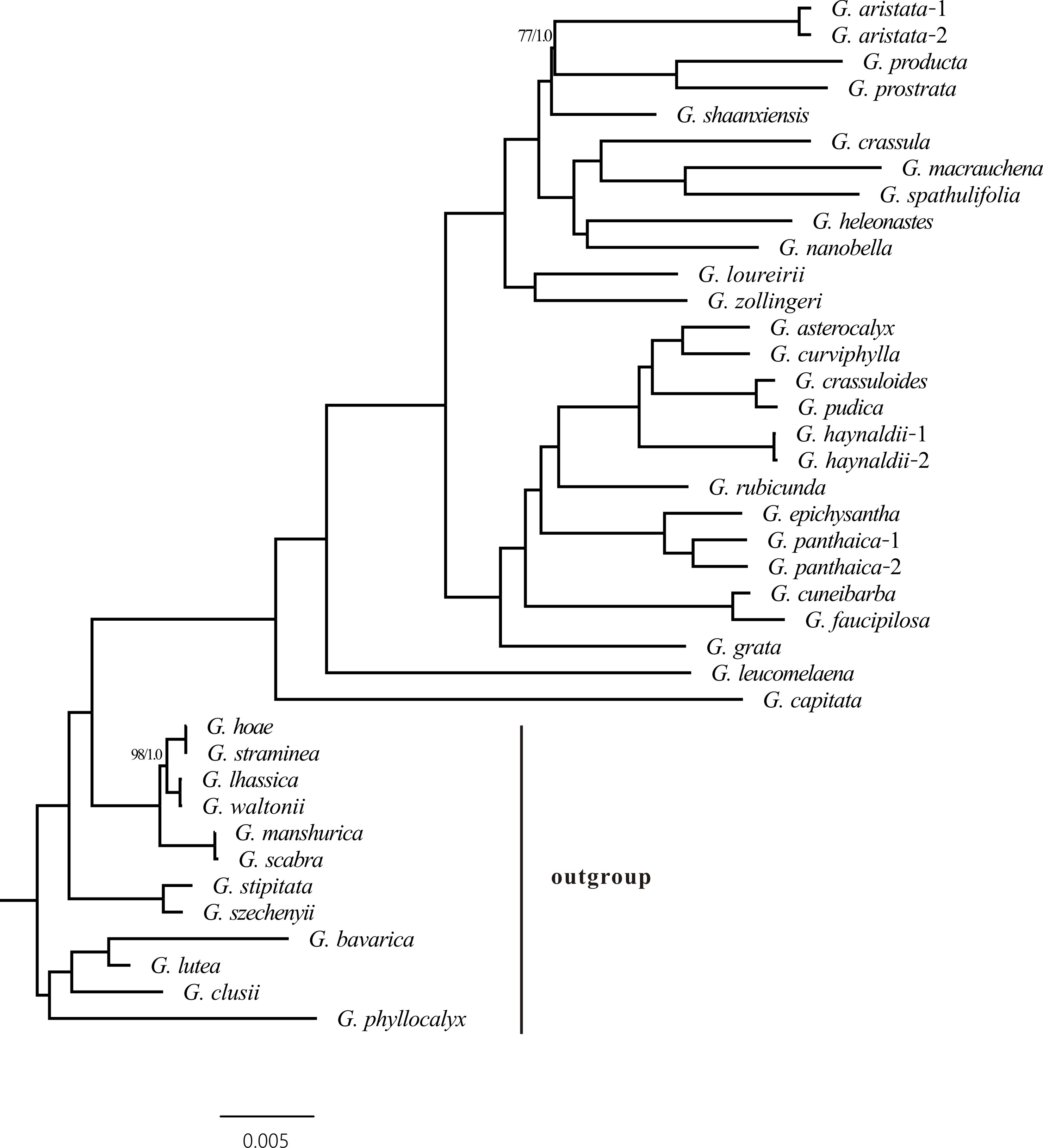

Supplement: Supplementary file 3 — Figure S1 [file ECE3-12-e9205-s004.jpg]

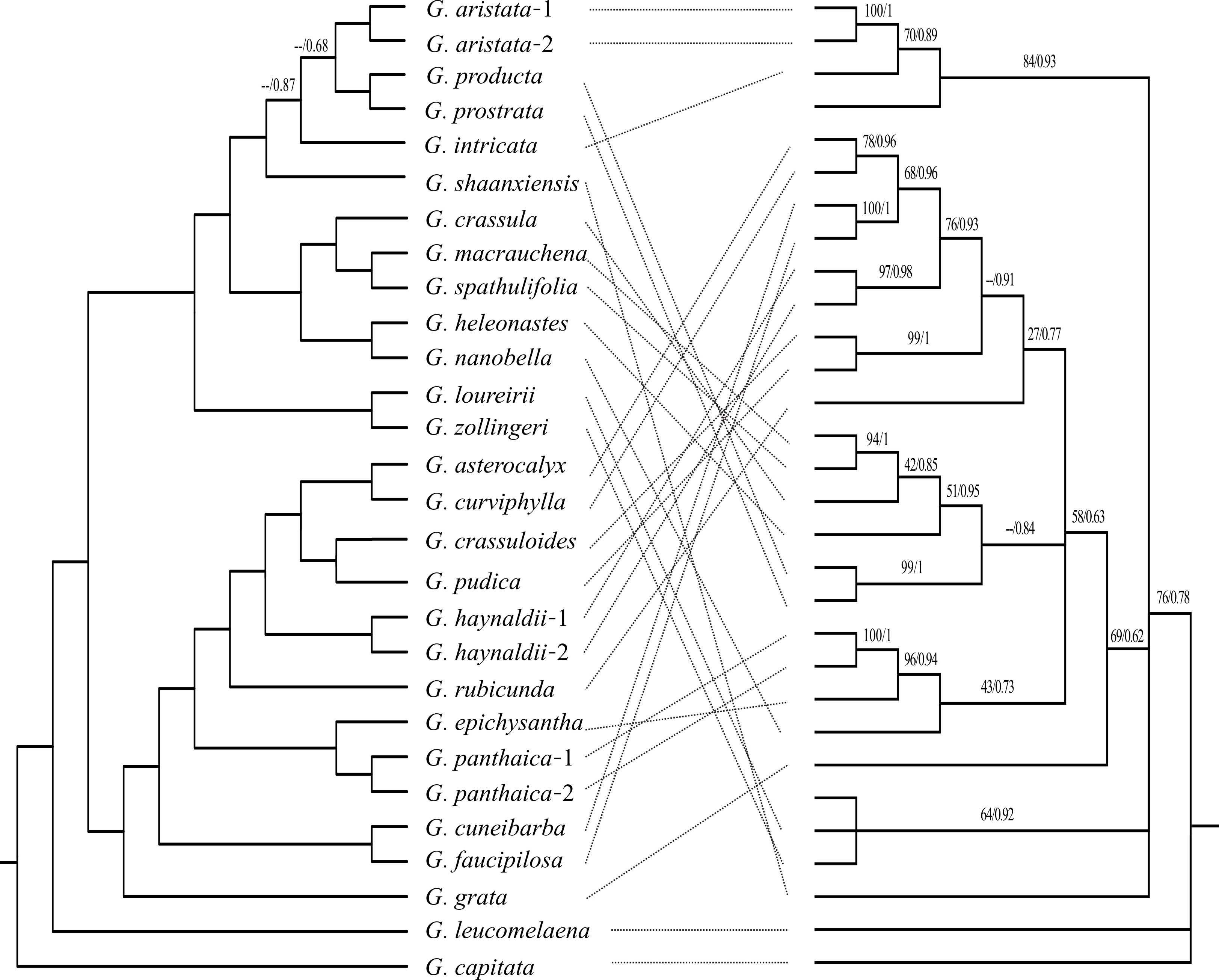

Supplement: Supplementary file 4 — Figure S2 [file ECE3-12-e9205-s003.jpg]

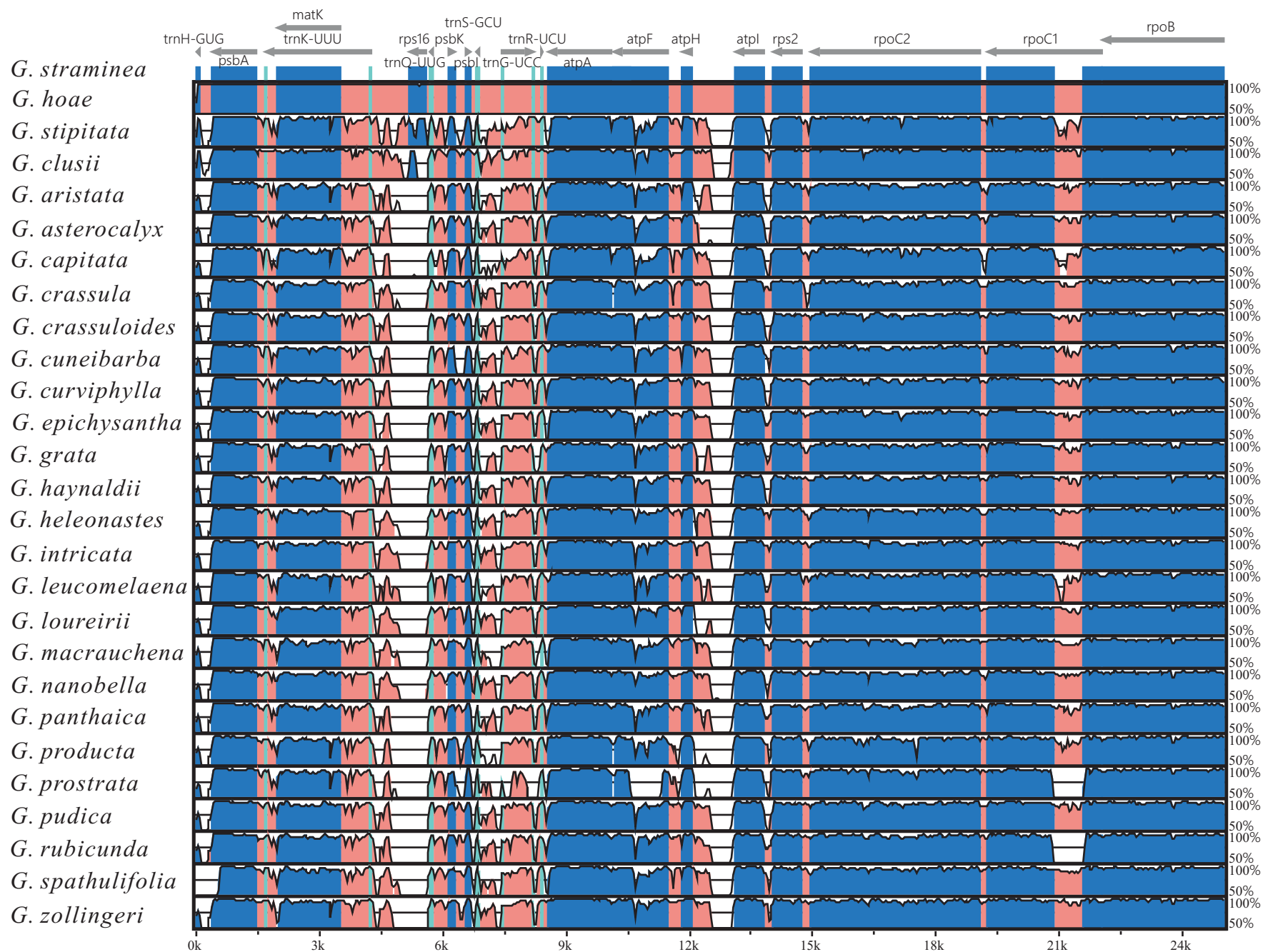

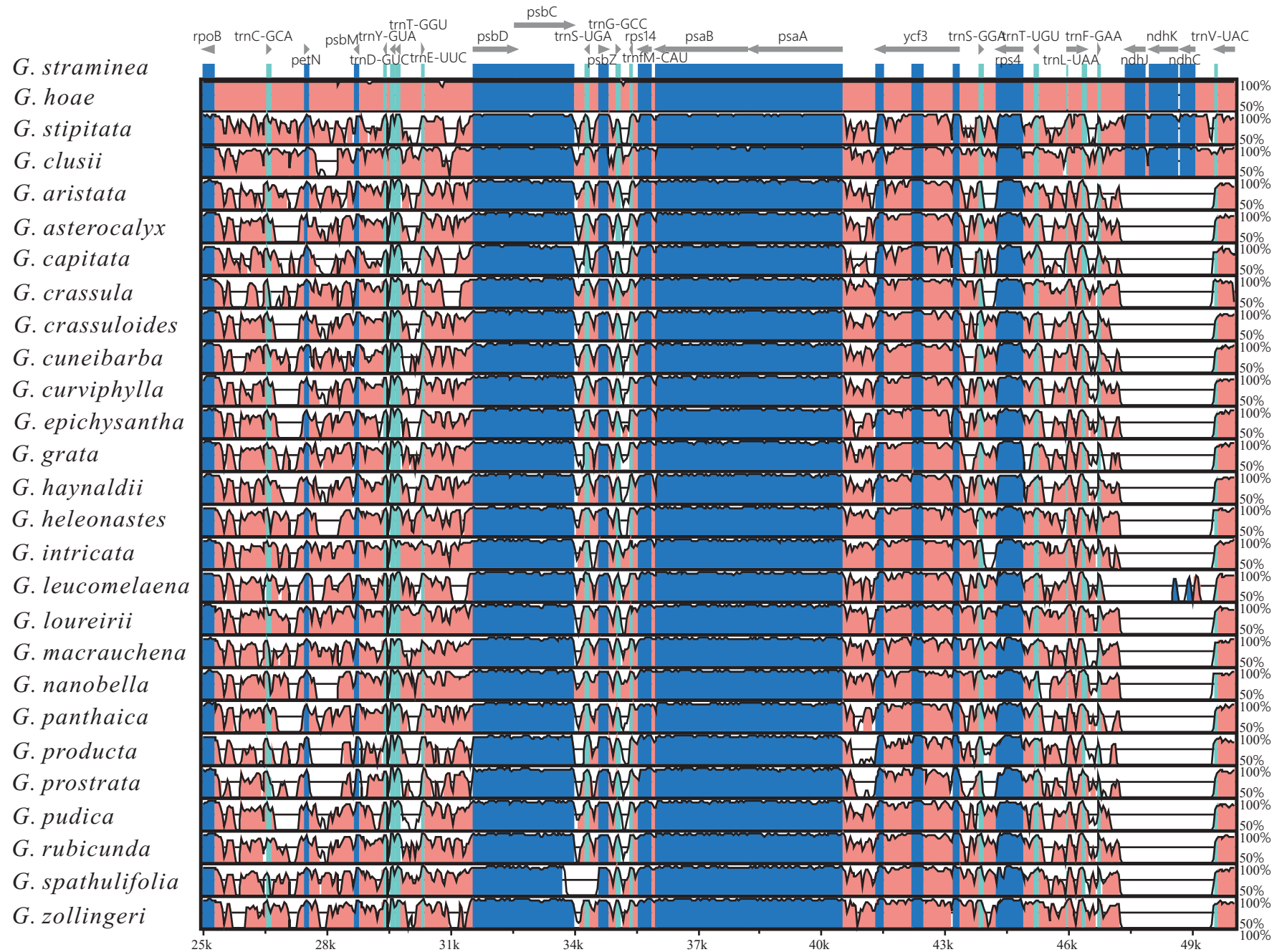

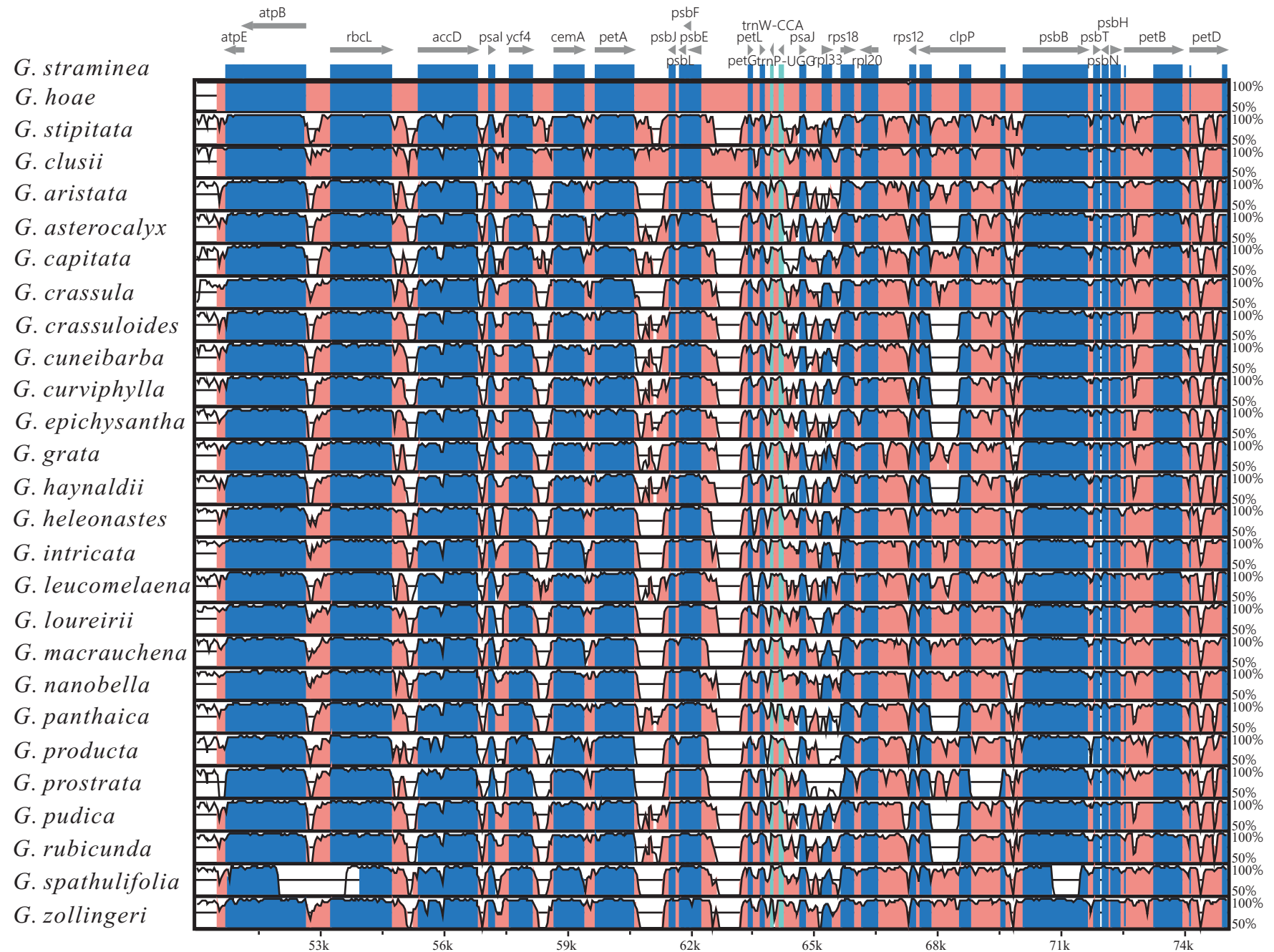

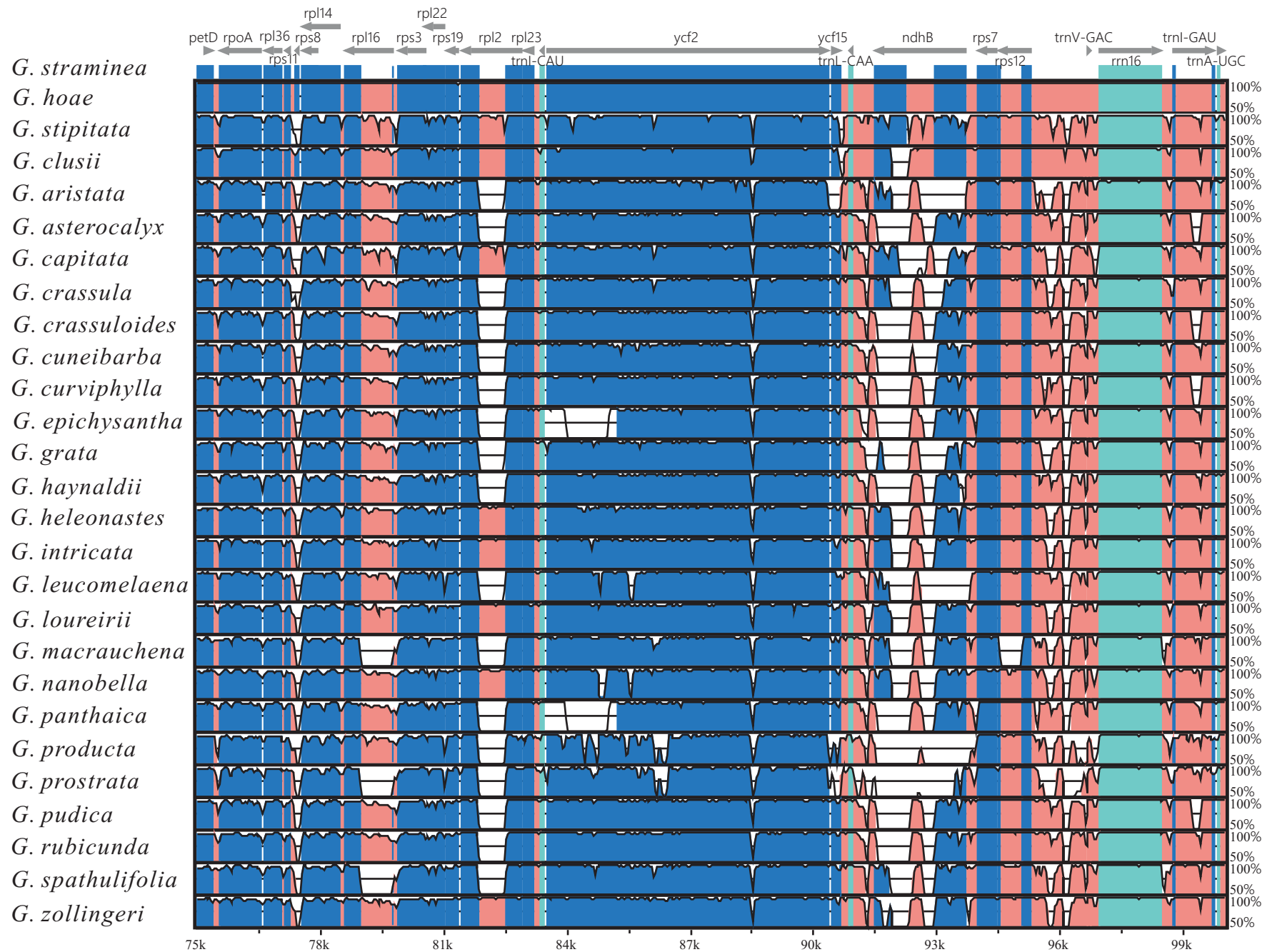

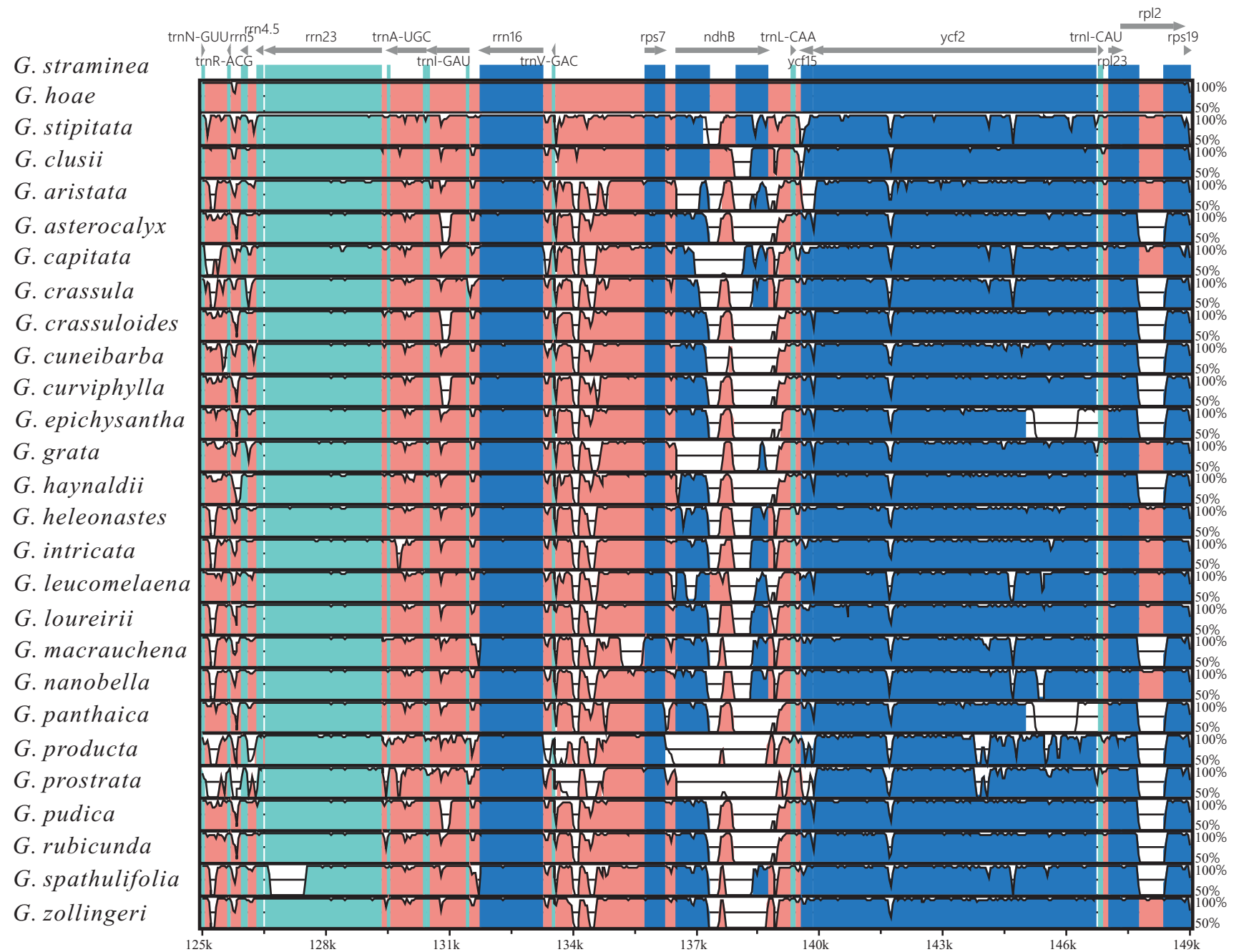

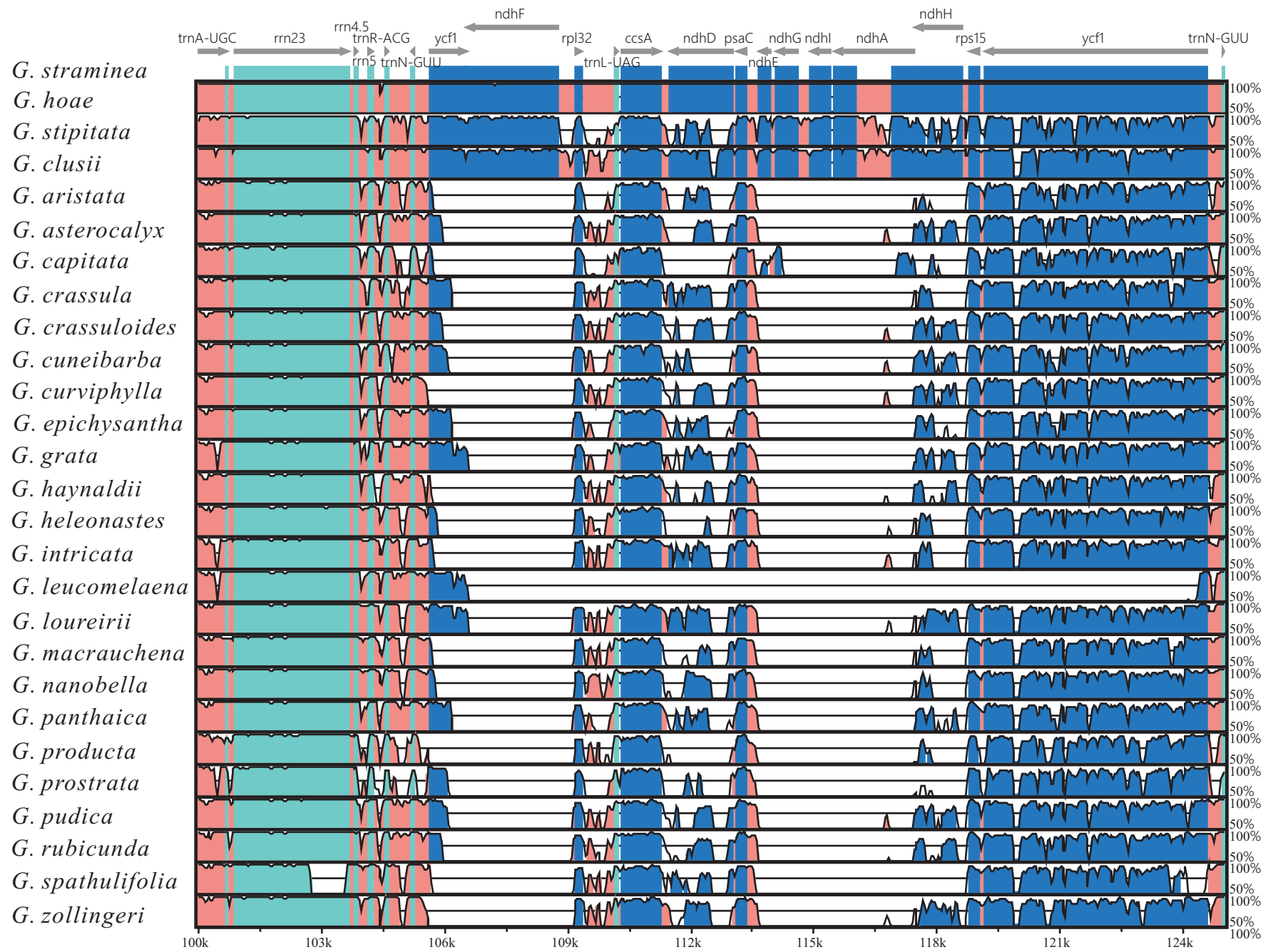

Supplement: Supplementary file 5 — Figure S3 [file ECE3-12-e9205-s002.pdf]
